# Editorial: Evolution and genomics of the *Mycobacterium tuberculosis* complex

**DOI:** 10.3389/fmicb.2023.1157559

**Published:** 2023-03-03

**Authors:** Ana M. S. Guimaraes, Adrian R. Allen, Marian L. Price-Carter

**Affiliations:** ^1^Department of Microbiology, Institute of Biomedical Sciences, University of São Paulo, São Paulo, Brazil; ^2^Department of Comparative Pathobiology, College of Veterinary Medicine, Purdue University, West Lafayette, IN, United States; ^3^Veterinary Sciences Division, Agri-Food and Biosciences Institute, Bacteriology Branch, Belfast, United Kingdom; ^4^AgResearch Ltd., Palmerston North, New Zealand

**Keywords:** *Mycobacterium tuberculosis* complex, genomics, evolution, *Mycobacterium bovis*, sequencing

## Introduction

The organisms that cause tuberculosis are responsible for debilitating, chronic disease across a wide swathe of the human and non-human animal kingdoms, with multiple host species affected by various ecotypes of the *Mycobacterium tuberculosis* complex (MTBC). These bacilli appear to exhibit a spectrum of host tropisms, with *M. tuberculosis* appearing to be a specialist human pathogen, while other members exhibit apparently broader host ranges across a range of livestock and wildlife that can facilitate zoonotic spread, adding to the human burden of disease (Malone and Gordon, 2017).

In terms of human health alone, there have been 10 million new cases per year and a fatality rate of ~1.3 million people per annum (WHO, 2022). As the SARS-CoV-2 pandemic starts to retreat, tuberculosis is regaining its position as the world's preeminent pathogen, worsened by an increased mortality rate due to healthcare disruptions caused by the pandemic (WHO, 2022).

The burden of tuberculosis in wildlife and livestock is not so well-documented, but in countries with established veterinary surveillance systems, it is recognized that multihost infections in wild fauna and domestic animals are common, leading to difficulties in eradication even when well-funded, state-led control schemes are in place. The zoonotic potential of these animal infections varies depending on global location, but it is undoubted that in many developing nations, the animal origins of human tubercular infections present a significant problem (Olea-Popelka et al., 2017).

Our understanding of the MTBC has been aided by historic molecular biological investigations that have revealed the genetic homology of its member ecotypes (>99.99% sequence identity over homologous regions), its clonal evolution, and its susceptibility to population bottlenecks and drift owing to its clonality. More recently, however, the advent and widespread use of genomic technologies have greatly aided our understanding of these and other facets of MTBC evolutionary biology. Specifically, genomic data are the highest resolution data available for any pathogen, and for the MTBC, they are revealing more regional and global diversity than was previously thought to exist. Such insights have benefitted epidemiological investigations, the detection/characterization of mechanisms of antimicrobial resistance, and a wider appreciation of the subtle heterogeneity across the MTBC that may have evolutionary consequences. As we improve our understanding of the MTBC's diversity and evolution, there will be tangible benefits for the control and eradication of these bacilli.

In this Research Topic, we sought to assemble a range of articles from across the world that used genomic methods and downstream analyses to characterize members of the MTBC, providing novel insights into the evolution of these bacilli.

In total, despite the effects of the SARS-CoV-2 pandemic on the academic, veterinary, and public health spheres, we received five papers from 19 authors from five countries (Ethiopia, France, Norway, Spain, and the United States of America). We summarize the key findings from these international submissions below, in chronological order of publication. We thank the authors for their contributions and trust that readers will find their work as stimulating to read as we found them to edit.

## Main scientific contributions of the articles composing this Research Topic

The study by Hakim and Yang analyzed the tertiary structure of the PPE18, the target protein of the M72/AS01 vaccine currently in a phase 2b clinical trial. By using 30 *in silico-*modeled PPE18 structures of *M. tuberculosis* strains from the USA and Turkey, the authors showed that PPE18 is composed of two domains with distinct variability profiles. The second domain varies in sequence and structure, which modulate the propensity of T-cell and B-cell epitopes. While T-cell epitopes are conserved among the analyzed orthologs, the likelihood of B-cell epitopes increases with sequence variability, possibly contributing to humoral immunity evasion. As humoral immunity against TB gains attention, these findings contribute to the understanding of the immunity induced by the M72/AS01 vaccine, and importantly, of how extant sequence variations can impact this immune response.

Welekidan et al. described, for the first time, the lineage diversity and antibiotic resistance mutations of *M. tuberculosis* from the Tigray region of Ethiopia using whole genome sequencing (WGS). Among 68 isolates, L4 and L3 lineages were predominant in Tigray; only one isolate each of L1 and L2 was detected. Comparing to other Ethiopian regions, the authors highlighted the great diversity of *M. tuberculosis* lineages in the country. While L4 showed higher proportions of drug resistance than L3, a great proportion (16%) of disputed RIF resistance mutations were identified and failed to be detected by an LPA (line probe assay). Therefore, the authors concluded that is urgent to implement a regional WGS facility to monitor drug resistance mutations in Ethiopia.

Two of the published articles, by Comín et al. and Charles et al., evaluated the insertion sequences (IS) *6110* of the MTBC. A major challenge in the analysis of the IS*6110* is to perform sequence comparisons that are locus-specific. By using a combination of WGS and Sanger sequencing of selected IS*6110* sites, Comín et al. explored locus-specific genetic variabilities of IS*6110* elements of the MTBC. They found that 13% of the IS*6110* copies had a mutation, reaching 31% when considering only LCN (low-copy number) strains. Their findings underscore the need to understand how these mutations affect IS transposition and consequently the IS-driven disruption of genes in the MTBC.

Strains of *Mycobacterium bovis* have been mostly found to harbor one or few copies of the IS*6110* (Gonzalo-Asensio et al., 2018). The finding of 11 IS*6110* copies in a French field strain (*M. bovis* Mb3601) (Branger et al., 2020) prompted Charles et al. to explore the copy number and insertion sites of the IS*6110* in *M. bovis* strains representative of the French genotype diversity. Among 81 *M. bovis* strains, 25% had between two and five copies and 10% had more than six copies of the IS*6110*. These high-copy strains (7–16 copies) were part of a monophyletic clade of the Eu3 clonal complex, which includes two important genotypes (SB0120-CO and SB0120-DHV). Within the SB0120-CO genotype, the authors showed that the IS*6110* sites are mostly stable over time and among host species. Since these multicopy *M. bovis* strains are circulating in French regions where bovine TB is most prevalent, the authors suggested future work should evaluate the influence of the IS*6110* on bacterial fitness and the epidemiological success of such strains.

Legall and Salvador leveraged robust datasets of *M. bovis* genomes of multihost systems from the USA, New Zealand, and the UK to identify SNP-dense and selective sweep regions that are important for the ecological adaptation of *M. bovis*. Using probabilistic frameworks within comparative genomics, the authors identified 14 SNP-dense regions (SDRs) and 132 selective sweep regions (SSRs) in 700 *M. bovis* genomes. Next, they measured the ability of these regions to differentiate isolates based on ecological grouping using random forest models. The accuracy of the models was higher when classifying the country of origin and subpopulation than the host species, but at the local level the model could segregate between cattle and wildlife in the UK with 88% accuracy. SSRs were better predictors of model classification than SDRs and affected genes of virulence and immunogenicity that might play a role in *M. bovis* adaptation to new environments. This study is one of the first to integrate comparative genomics and machine learning to understand the evolution of *M. bovis*, which sets a mark on how sophisticated future analysis will be in the research field.

## Conclusion

This Research Topic was frustrated somewhat by the SARS-CoV-2 pandemic, whose disruptive effect on the normal progression of science was substantial. Many authors who expressed an interest in contributing to the topic were unfortunately unable to participate, owing to other commitments over the period of 2020–2022. Despite this, the work presented here is testament to the ongoing effort to understand the MTBC better using genomics.

A feature of the MTBC, aided by its clonality, is its tendency to evolve lineages with specific geographical associations and, potentially, region-specific phenotypes. In the past, our knowledge of the complex was often based on a few well-defined lineages/strains from Western and Anglophone countries, leading to generalizations based on fundamentally biased datasets. As we look more closely at the MTBC from all regions of the world, we find more heterogeneity than was originally proposed to exist. Knowledge of such heterogeneity and its possible consequences is important, as it can provide a means to infer infection sources in our increasingly globalized world, help identify novel mechanisms of antibiotic resistance, and lead to novel hypotheses about MTBC evolution and possible pathogen phenotypes.

We believe this observation should lead us to embrace a more outward-looking, international view of this group of pathogens. Greater collaboration across borders can only enhance our knowledge further and lead to better control and eradication strategies.

## Author contributions

All authors listed wrote and reviewed this editorial, making substantial contribution to the work, and approved it for publication.
